# Text Detection Using Multi-Stage Region Proposal Network Sensitive to Text Scale †

**DOI:** 10.3390/s21041232

**Published:** 2021-02-09

**Authors:** Yoshito Nagaoka, Tomo Miyazaki, Yoshihiro Sugaya, Shinichiro Omachi

**Affiliations:** Graduate School of Engineering, Tohoku University, Sendai 9808579, Japan; naga.yoshi.yoshi@gmail.com (Y.N.); sugaya@iic.ecei.tohoku.ac.jp (Y.S.); machi@ecei.tohoku.ac.jp (S.O.)

**Keywords:** scene text detection, multiple scales, convolutional neural networks

## Abstract

Recently, attention has surged concerning intelligent sensors using text detection. However, there are challenges in detecting small texts. To solve this problem, we propose a novel text detection CNN (convolutional neural network) architecture sensitive to text scale. We extract multi-resolution feature maps in multi-stage convolution layers that have been employed to prevent losing information and maintain the feature size. In addition, we developed the CNN considering the receptive field size to generate proposal stages. The experimental results show the importance of the receptive field size.

## 1. Introduction

Recently, attention has surged concerning intelligent sensors using text detection [1,2]. Texts in a natural scene image are useful for many applications, such as translator, mobile visual search, and so on. Thus, text detection is a hot topic in computer vision. A convolutional neural network, CNN, is widely used in object detection tasks since its high performance. Particularly, Faster R-CNN [3] is a standard method. Moreover, there are YOLO [4,5,6] and SSD [7]. Text detection benefits from CNN-based object detection to achieve high performance.

**Figure 1:**
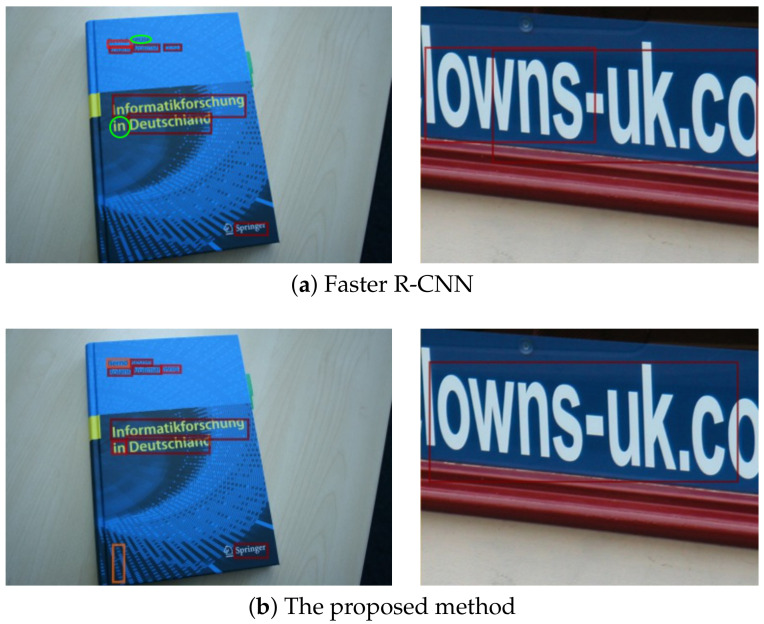
Detection examples. (**a**) Faster R-CNN (convolutional neural network) failed to detect small texts and detected large texts only partially. Green circles are the missed texts. (**b**) The proposed method detected small and large texts successfully. Although, there is the false-positive detection in the left example.

It is unsuitable to directly apply object detection methods [3,4,5,6,7] to text detection. As shown in Figure 1a, the small texts “reuse” and “in” were missed in the left example, and large text “lowns-uk.co” was divided in the right example. The CNNs transformed images into a low-resolution feature maps. Thus, some texts are transformed to appropriate scales in the feature maps. However, small and large texts became inappropriate scales, resulting in detection failures.

There is room in Faster R-CNN to improve scale sensitivity. Its limited scale sensitivity is due to a fixed receptive field in region proposal network (RPN), a Faster R-CNN module. RPN extracts context features around objects using convolutional computation. The receptive field size of the RPN is essential. The convolutional computation produces one pixel in a feature map from a fixed area context. For example, 3×3 kernel of the convolution produces one output from 3×3 input. The receptive field depends on the number of convolutional computations. In the case of Faster R-CNN, the receptive field is 228×228. We doubt whether Faster R-CNN can utilize the context information well because it has a fixed receptive field.

The problem of small object detection is caused by detection from only one feature map. Recently, multi-stage convolutional feature maps [8,9] are applied to many works for not only object detection but also other tasks. While this strategy is useful, but there are few discussions about quantitative analysis. He et al. [10] introduced a skip-connection module to prevent overfitting, which was the first attempt to merge different feature maps. Wang et al. [11] explained the effectiveness of using convolutional layers simultaneously. These explain the effectiveness of using multi-feature maps; however, there are no detailed works on the receptive field, to our knowledge. Our proposed idea computes the receptive field size. Therefore, it can extract adequate context features for generating proposals. Besides, the proposed idea can be applied to other detection modules and tasks.

To reinforce the scale sensitivity, we propose a CNN that can detect small and large texts simultaneously. Specifically, we propose to use multiple RPNs to generates text proposals in different resolution feature maps. These multiple RPNs have different receptive field sizes. As shown in Figure 1b, the proposed method detected small and large texts successfully. The contribution of this paper is the integration of Faster R-CNN and a multi-resolution detection approach using multiple anchors of the appropriate dimensions for texts. The proposed architecture is sensitive to text region scale by using a multi-receptive field size. We confirm that the receptive field is an important factor when using the CNN, and the proposed concept can contribute to other detection methods.

This paper is an extended version of our conference paper [12]. There are four differences from the conference paper. Firstly, we reorganized the related work section using more than 20 additional literature to clarify the background of the proposed method. Secondly, we conducted an ablation study to confirm improvements of the two proposed components, multiple RPNs and Anchor. Section 4.3 summarizes the results. Thirdly, we visualized the output of each RPN to confirm output scales are appropriate. Section 4.4 showed that text detection is performed by RPNs that are responsible for small and large scale, respectively. Finally, we analyzed failure results by investigating the output of the RPNs and activated feature maps. Section 4.5 illustrated the output. Overall, these four additional discussions and experiments reinforced the conference paper.

## 2. Related Works

A text detection method is based on object detection. Hence, we describe object detection methods. Then, we address some studies to use multi-resolution feature maps for object detection. Finally, we introduce text detection studies.

### 2.1. Object Detection

Object detection is a popular research subject in computer vision. There have been many attempts, such as deformable part model [13] and histograms of sparse codes [14] which use engineered feature expression and support vector machine. These methods incur high computation cost because they need many feature expressions and parameters for evaluation. Recently, the CNN-based method and R-CNN [15] have been used for object detection. R-CNN is composed of a proposal generation stage and a classification stage. Proposals from a given image are generated using modules of other methods such as Selective Search [16]. The proposal regions cropped from an original input image are fed into the classification stage, which uses the CNN to classify proposals into the object or background classes. In addition, the bounding-box regression process adjusts the proposal rectangles to object sizes accurately. The problem of R-CNN is high computation cost because the CNN computes the feature map for each proposal. In the Fast R-CNN [17], RoI-pooling (region of interest pooling) is introduced to share precomputed convolution features. Given an input image, the CNN computes the feature maps of the whole image. The feature maps of the proposal regions are cropped and pooled to the fixed size by using RoI-pooling. This reduces the computation cost; however, the Fast R-CNN requires another pipeline to generate proposals. Therefore, it cannot process end-to-end consistently. The Faster R-CNN [3] uses the RPN to generate proposals with only convolutional layers. In the RPN, the convolutional layer (3×3 kernel) is applied to obtain the feature map, which is fed into two sibling convolutional layers (1×1 kernel) for binary classification (object/background) and bounding-box regression. In each pixel position of the feature map, some proposals with confidence scores are generated from fixed-size rectangles called anchors in the bounding-box regression. Therefore, the Faster R-CNN does not require an external proposal generating method by RPN module. The Faster R-CNN is a baseline method for achieving state-of-the-art accuracy and inference speed. This realizes end-to-end processing and improves the detection speed and accuracy.

YOLO (you only look once) [4,5,6] is a one-shot detector and is not a region-based method. It predicts proposals with object likelihood scores and class probabilities. Therefore, it does not need any computation modules per proposal. This leads to less computation than the Faster R-CNN. SSD (single shot multibox detector) [7] is similar to YOLO, except for using a multi-resolution feature map for detection. SSD predicts the proposals from each convolutional layer. Therefore, it has various features for detection, unlike the Faster R-CNN and YOLO.

### 2.2. Strategy Using Multi-Features

The CNN is composed of many convolutional layers, e.g., 13 layers in VGG16 [18]. In general, a shallow layer extracts simple features of an image, called as a low-level feature, and a deeper layer can extract complex features, called as a high-level feature. Therefore, many works using the CNN use many convolutional layers. However, using many convolutional layers incurs high computation cost. To avoid this, a downsampling operation called pooling is inserted after some convolutional blocks; however, it leads to loss of feature information as a trade-off. Many recent works have pointed out this phenomenon, particularly in object detection, face detection, and text detection.

A recent trend of using multi-stage convolutional feature maps is called feature pyramid. Kong et al. [9] pointed out that region-based methods struggle with small-size objects. To solve this problem, they use conv1, conv3, and conv5 feature maps of VGG16 and merge them into one feature map. This generates large-size feature maps using multi-feature states. Kong et al. [19] merge the convolutional feature map and deeper feature for accurate object localization. Lin et al. [8] applied feature merging to the Faster R-CNN and concluded that using feature hierarchy saves memory cost. Wang et al. [11] used a multi-convolutional layer for high-order statistics to represent feature maps with negligible computation cost.

These strategies are inspired by skip-connection [10], and it leads to semantic segmentation [20,21,22] along with detection. In this work, we also considered receptive fields of the multi-stage convolutional layer.

### 2.3. Text Detection

Text detection has been widely studied for decades. Wang et al. [23] detected characters using the sliding window and random ferns [24] and connected the characters using pictorial structures [25]. Wang et al. [26] detected word regions using the sliding window and CNN, and recognized characters using the CNN and dictionary-matching. Milyaev et al. [27] binarized images and generated word proposals integrated from connected components by edge information and engineered features such as position and color. The character proposals classified by AdaBoost were connected to word proposals, which were followed by recognizing the word proposals by OCR (optical character recognition). Opitz et al. [28] generated a text region confidence map using the sliding window and AdaBoost, and detected word regions by maximally stable extremal region [29]. After detection, they recognized the text using CNN from a pre-defined dictionary. Jaderberg et al. [30] used edge boxes [31] and aggregate channel features detector [32] to generate text proposals and eliminate false positive proposals using random forest. Then, they used the CNN for bounding-box regression and recognizing characters. Tian et al. [33] generated character proposals using the sliding window and fast cascade boosting algorithm [34] and connected the characters using the CNN. These methods involve multi-stage processing and complex pipeline. Hence, they require fine parameter tuning for generating proposals and classifying them. Recently, the deep learning approach has been frequently used because it does not require engineered features. In addition to this, the CNN-based detection approach involves a simple architecture, realizing end-to-end consistent flow without complexity.

Therefore, many approaches are based on the recent progress in the end-to-end process of object detection. Liao et al. [35] proposed end-to-end CNN-based SSD, employing a horizontally long anchor to detect the text region efficiently. SSD uses multi-stage convolutional feature maps. Therefore, this approach is close to our proposed method. Tian et al. [36] predicted parts of the text region using the RPN to predict vertically long proposals having fixed widths. The proposals are connected by bi-directional LSTM (long short term memory), and the final output is the bounding-boxes of the text regions. Zhong et al. [37] improved the Faster R-CNN for text detection. By introducing an inception module [38], they used convolutional operations having multi receptive field and this leads to extract features efficiently compared with the conventional convolutional layer.

Recently, segmentation-based approaches are often employed. Tang et al. [39] used three CNNs for text region segmentation: One predicts the text region roughly, the second one refines the text region pixels, and the last one judges whether the text region is correct or not. Dai et al. [40] combined the Faster R-CNN and segmentation for arbitrary-oriented text. This predicts the text mask after generating the proposals. Lyu et al. [41] predicted position-sensitive segmentation, which is robust to arbitrarily inclined text positions. Zhou et al. [42] proposed segmentation- and parameterize-inclined text region by expressing the distance from the pixels. Bounding-boxes were generated based on the distance from one pixel in the text mask. This approach has simple architecture and can predict arbitrary coordinates of the bounding-boxes. He et al. [43] also predicted the parameters of the relative positions for the bounding-boxes using segmentation strategy with fully convolutional network.

Not only text detection but also recognition methods are studied for recognizing words by CRNN (convolutional recurrent neural networks) [44] using connectionist temporal classification loss [45]. Bušta et al. [46] predicted the text region using an anchor-based detector such as Faster R-CNN, and each region was recognized using the CRNN. Li et al. [47] combined the LSTM with the Faster R-CNN to realize text spotting (detection and recognition). First, the Faster R-CNN block outputs text bounding-boxes, and the two LSTMs, encoder LSTM and decoder LSTM, recognize the word in the bounding-box. This method detects text and recognizes end-to-end consistently using one deep learning model. Liu et al. [48] also combined text detection and recognition. In the text detection stage, this predicts arbitrarily oriented regions such as [42]. In the recognition stage, the proposals are rotated by affine transformation and are inputted in the CRNN module containing bi-directional LSTM and outputs labels.

Thus, the text detection methods have progressed notably in the virtue of CNN. We applied Faster R-CNN for object detection because this can be expanded to many works and be used as a baseline.

## 3. The Proposed Methods

In this section, we describe the proposed CNN module and its core concept.

### 3.1. Scale-Sensitive Pyramid

**Figure 2:**
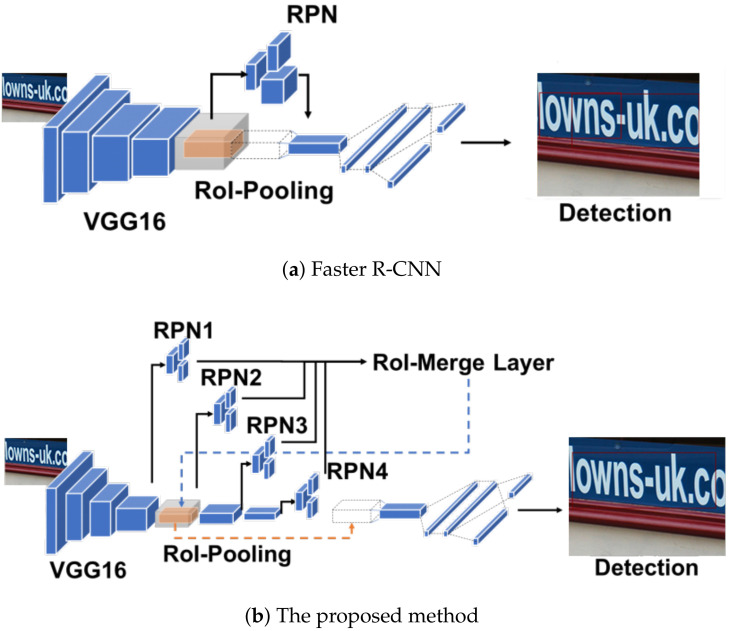
Architectures.

The proposed architecture is depicted in Figure 2. The main difference between Faster R-CNN and the proposed method is the total number of RPNs. While Faster R-CNN has one RPN in conv5-3 of VGG16, the proposed method has four RPNs in each convolutional layer. Specifically, RPN1, 2, 3, and 4 are added to conv4-6, conv5-3, conv6-3, and conv7-3, respectively. To use a large receptive field in the proposed architecture, we added two convolutional blocks containing one max-pooling and three convolutional layers such as VGG16. In addition, we used deep-feature representation in the conv4 stage and added extra three convolutional layers after conv4-3.

In this paper, we define the number of RPNs as four to consider two purposes. Firstly, we aim to maximize two evaluation metrics, Recall and Precision. There is a trade-off between them. We can obtain a higher recall value by increasing the number of RPNs since more RPNs produce more text candidates. In contrast, the precision value decreases as the number of candidates increases. Thus, we determined the number of RPNs heuristically by considering the trade-off. Secondly, we aim to make training stable and feasible. There will be more training parameters when the number of RPNs increased. Consequently, training will be unstable. Besides, the amount of GPU memory is limited. Therefore, four is a feasible amount of RPNs for training. Although there is no experimental support, the above purposes are based on general facts. The trade-off between recall and precision is widely known. Moreover, training may be unstable if learning parameters increased. Thus, we believe the reasons are convincing.

The RPNs generate proposals using each pixel of the feature maps. Thus, the proposals were largely influenced by the convolutional layers. The convolutional layer having 3×3 kernel gathers 3×3 the size context in the input feature map to one pixel as the output. Therefore, two accumulated convolutional layers gather 5×5 the context to one pixel. Considering this for an input image, we can determine the context size in the input image, which influences the generation of proposals in the RPN. In this paper, we denote this context size as a receptive field. The RPN of Faster R-CNN has a 228×228 size of the receptive field. However, it is not sufficient to obtain information for detection, considering that the input size is about 600×600. On the other hand, the proposed method has four RPNs, which have various receptive fields. The receptive fields of the RPN1, 2, 3, and 4 are 156×156, 228×228, 468×468, 948×948, respectively. Therefore, while RPN1 can use fine context to generate tiny proposals, RPN3 and RPN4 can use a large context to enclose large text. We call this proposed architecture SSP-RPNs (scale-sensitive pyramid RPNs) for convenience.

The SSP-RPNs have more RPNs than Faster R-CNN. Therefore, we introduce an RoI-merge layer to prevent the increase in the computation cost for the proposals. The RoI-merge layer receives 400 proposals (each RPN outputs 100 proposals) and applies non-maximum suppression to eliminate the overlapped proposals. Then, it selects up to 100 proposals by a higher confidence score as output proposals.

### 3.2. Anchor for Text Detection

Anchor is rectangular with a fixed size in the RPN, and this is regressed to arbitrary-sized nonlinear transformation called bounding-box regression. However, transformation parameters are determined from anchor’s height or width. Hence, the proposals are mainly dependent on the anchor. Thus, we need to select an efficient anchor size for text detection. The main target of this work is Latin scripts containing alphabets and digits, and we can consider Latin scripts to be horizontally long instead of vertically long.

**Figure 3:**
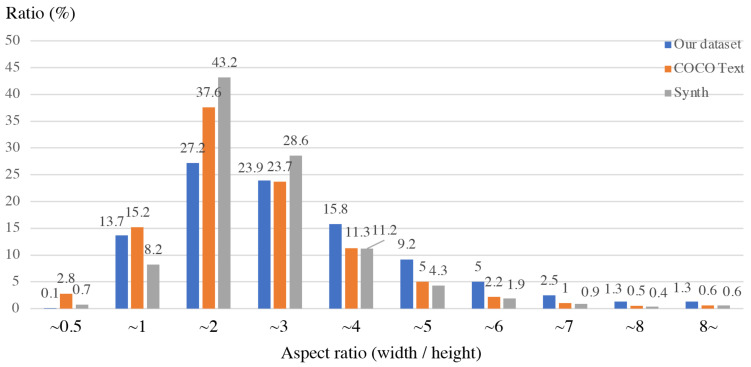
Histogram of the aspect ratio of texts in Datasets.

**Figure 4:**
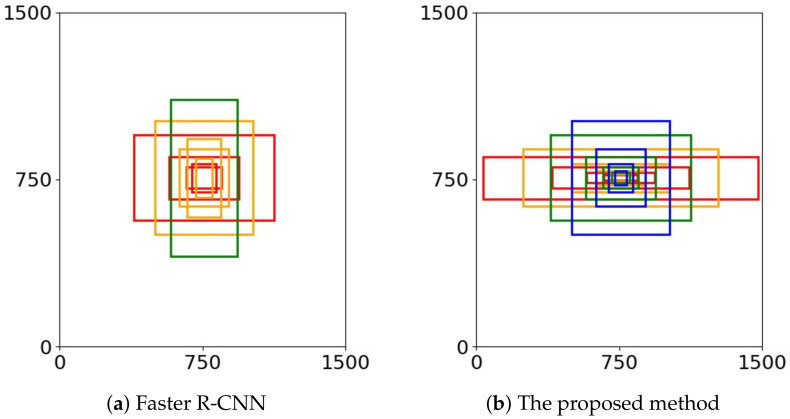
Comparison on anchors.

First of all, we performed the statistics for the text sizes in natural scene images. Figure 3 shows that the histogram result of the aspect ratio (width/height) in three training datasets: COCO Text [49], Synth Text [50], and our dataset described in Section 4.1. The reliability of the histogram is based on diversity in the datasets. Specifically, our dataset is composed of five public datasets, which are widely used in text detection studies. Furthermore, COCO Text and Synth Text are large datasets containing 173 K texts and 8 M words, respectively. The histograms shows that the text bounding-boxes are horizontally long, and particularly the half of them have widths two to four times the height. Faster R-CNN prepares various anchors depicted in Figure 4a. It contains horizontally long and vertically long aspect ratios of 1:2, 1:1, 2:1. Considering the statistics of the text bounding-boxes, a vertically long anchor is unnecessary, and we need more horizontally long anchors. In addition to this, the demand for a small-scale anchor increases because of the smallest receptive field size of the SSP-RPNs module of 156×156.

Based on the above reasons, we proposed new anchors for text detection shown in Figure 4b. We eliminated vertically long anchors and added horizontally long anchors for the Latin text. Moreover, we added a small-scale anchor for tiny text. For large-scale text, Faster R-CNN prepares large scale anchors, and we do not add any large-scale anchors. In the experiments, we confirmed that the proposed anchors were more efficient than the default anchors, and the anchor was an important factor for generating proposals.

### 3.3. Training Strategy

The total loss for the proposed method is Equation (1).

(1) $$L_{total}=\sum_{i\in \{1,2,3,4\}}\lambda_{i}L_{rpn_{i}}+\lambda_{fastrcnn}L_{fastrcnn}$$

$L_{rpn_{i}}$ represents the loss of each RPN, and $L_{fastrcnn}$ is the loss of Fast R-CNN. $\lambda_{*}$ means the hyper parameter to define the loss balance, we set $\lambda_{*}=1$ in the experiments. $L_{rpn_{i}}$ and $L_{fastrcnn}$ are composed of the classification loss and bounding-box regression loss, respectively. Detailed explanation can be found in [3,17].

We assign ground-truths to RPNs according to their sizes. Let ground-truth’s size be maximum of either height or width. RPN1 is responsible for sizes less than 140. Followed by [3], RPN2 undertakes all ground-truths. Both of RPN3 and RPN4 take responsibility for sizes larger than 220. Overall, RPN1 is trained to be suitable for small-scale text, RPN3 and RPN4 are used for large-scale text.

## 4. Experiments

In this section, we evaluated the proposed method and compared it with other text detection methods. In training, the proposed model’s parameters were initialized using ImageNet pretrained model, and the layers other than VGG16 were initialized according to Gaussian distribution (mean is 0, the standard deviation is 0.01). The learning rate was fixed to 0.001, weight decay was 0.0005, momentum was 0.9, and we iterated 100 K. For both training and testing, we used GPU NVIDIA TITAN X (Pascal). We implemented the proposed method using the faster R-CNN based on the deep learning framework, Caffe (Implementation of Faster R-CNN with Caffe: https://github.com/rbgirshick/py-faster-rcnn accessed on 29 December 2020).

### 4.1. Datasets and Evaluation Metrics

We compiled our training dataset including 7152 natural scene images containing texts. Our dataset is composed of five public datasets: ICDAR2013 RRC focused scene text training dataset (229 images) [51], ICDAR2015 RRC incidental scene text training dataset (1000 images) [51], ICDAR2017 RRC multi lingual text training dataset (5425 images) [52], street view text training dataset (SVT Dataset: http://vision.ucsd.edu/~kai/svt accessed on 29 December 2020), and KAIST dataset (KAIST Dataset: http://www.iapr-tc11.org/mediawiki/index.php/KAIST_Scene_Text_Database accessed on 29 December 2020) (398 images). We evaluated the methods on the ICDAR2013 RRC focused scene text test dataset (233 images).

We used DetEval [53] containing three evaluation protocols, recall, precision, and F-score. The Recall represents that how much ground-truth is covered by the detection results. Precision means that how accurately the methods generate the bounding-boxes. F-score is the harmonic mean between recall and precision.

### 4.2. Numerical Results

The numerical results are shown in Table 1. The full results are available online (Online results (Proposed): https://rrc.cvc.uab.es/?ch=2&com=evaluation&view=method_info&task=1&m=50094 accessed on 29 December 2020)

We compared the proposed method to other methods [3,33,35,37,43,50]. Particularly, Faster R-CNN [3] is an essential baseline of the proposed method. The fundamental difference is the number of RPNs: one in the Faster R-CNN, four in the proposed method. Using only one RPN struggles with detecting small and large texts. Therefore, we proposed to use four RPNs that are responsible for small and large texts, respectively. To verify the effectiveness of using four RPNs, a comparison with Faster R-CNN is necessary.

The proposed method outperformed Faster R-CNN more than seven points at F-score. Thus, we confirmed that the scale sensitivity could bring a certain improvement to text detection. Moreover, we showed the results of the proposed method in competition mode. The full results are available online (Online results (Proposed, Competition mode): https://rrc.cvc.uab.es/?ch=2&com=evaluation&view=method_info&task=1&m=51720 accessed on 29 December 2020).

The comparison methods can be divided into two approaches in the aspect of scale strategy: multi-scale [35,43,50] and single-scale [3,33,37]. The multi-scale approach produces multiple resolution images using various scale ratios. A post-processing is required to merge results in multiple images. The single-scale approach uses a single resolution image and applies multiple-sized kernels to detect various scaled texts.

**Figure 5:**
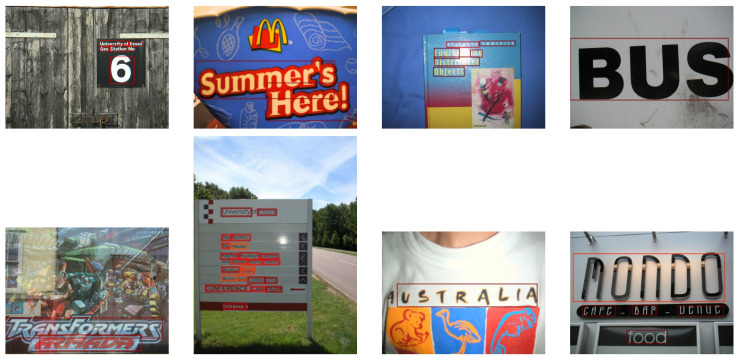
Result examples on ICDAR2013. Red rectangles are detection results by proposed method.

According to the numerical results, the multi-scale methods were superior to the single-scale methods. Especially, the results of [43] are better because of the number of input images, such as seven images by scale ratios, $2^{\left\{-5,\cdots ,1\right\}}$. The abundant input images are essential in the multi-scale approach. However, simultaneous detection for small and large texts is difficult in the multi-scale approach since small texts are collapsed easily. On the other hand, the proposed method keeps small and large texts intact. The multiple RPNs search texts in different resolution feature maps extracted from only one single image. As shown in Figure 5, the proposed method can detect various texts containing tiny-scale and large-scale texts.

### 4.3. Ablation Study

We discuss the effectiveness of the proposed method by ablation study. There are four variations. The first is baseline Faster R-CNN. The second is Faster R-CNN with the proposed anchors (Anchor). The third is Faster R-CNN with SSP-RPNs (SSP-RPNs). The last is the proposed method with the proposed anchors and SSP-RPNs (Proposed). We used the same environment and hyperparameters for training all the variations.

We showed the results in Table 2. Compared to the baseline and Anchor, F-score improved by 6 points, which indicates the effectiveness of the proposed anchors. The anchor is an important factor to generate bounding-boxes in the RPN. Compared to the baseline and SSP-RPNs, F-score improved by 1.5 points. We confirmed that the scale sensitive module made detection effective. The proposed method was better than other methods. Therefore, both the proposed anchors and modules should be robust for text detection. The proposed method also improved the precision with a large margin. Thus, the proposed method learned to generate proposals by reducing negative proposals. Overall, the proposed method improved robustness with the help of the multiple RPNs.

**Figure 6:**
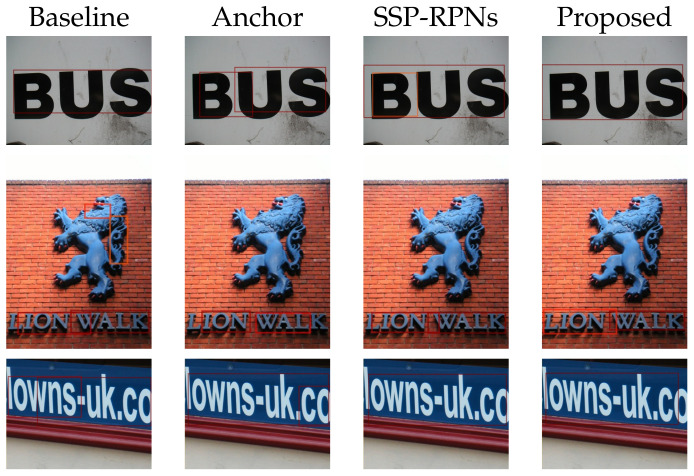
Detection examples in ablation study.

Subsequently, we discuss the detected bounding-boxes. Figure 6 shows that the proposed method can utilize the receptive field and context. On the other hand, the baseline failed to enclose the texts entirely. The RPN in the baseline has 228×228 receptive field, which is smaller than the target text scale. We assumed that this failure was due to less context. Compared to the baseline, the proposed method enclosed large-scale text completely. The large receptive field of the proposed method extracted enough context to confirm the existence of large texts in image. Consequently, we achieved accurate detection.

The third row in Figure 6 also shows the validity of Proposed. The anchor model detected large texts, however, they are partial. This failure was caused by a small context in target texts. On the other hand, the SSP-RPNs model and the proposed model detected large texts successfully. These results show that a horizontally long anchor is necessary for Latin text detection. Besides, receptive field positively contributes to generating proposals. Thus, SSP-RPNs module is essential.

### 4.4. Scale Sensitive Strategy

**Figure 7:**
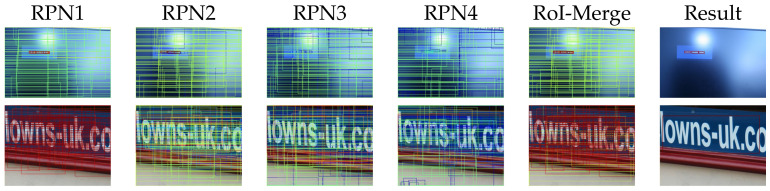
The outputs from each region proposal network (RPN). Red rectangles have high confidence, and blue rectangles have low confidence.

In this section, we evaluate the SSP-RPNs module. Figure 7 showed that the outputs of each RPN, RoI-merge layer, and results. The upper row in Figure 7 is a tiny-scale text case. The RPN1 generated proposals fitted to the tiny text with high confidence, whereas RPN3 and RPN4 failed. After the RoI-merge layer, the proposals of RPN1 were selected. Consequently, detection succeeded in the final result. These results verified that RPN1 learned small texts. The lower row in Figure 7 is a large text case. The proposals of RPN1 were too small to enclose the entire text region. Whereas RPN3 and RPN4 generated proposals enclosing the whole text region. Consequently, the large texts were detected in the final result.

Overall, each RPN learned to detect each suitable scale text corresponding to their receptive field sizes, i.e., RPN1 was optimized for small-scale, and RPN3 and 4 were optimized for large-scale. Therefore, these RPNs can help RPN2, which is in its original position after conv5-3. Moreover, the RoI-merge layer is necessary for the SSP-RPNs module to reject unnecessary proposals.

### 4.5. Failure Analysis

**Figure 8:**
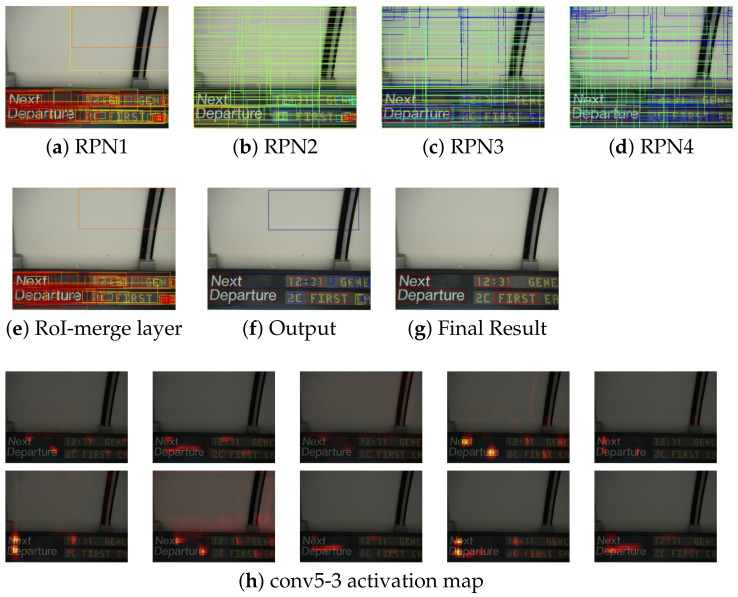
Failure examples 1. (**f**) classification results of the proposals. Red and blue represent text and background, respectively. Activation maps in (**h**) are resized to the input size.

**Figure 9:**
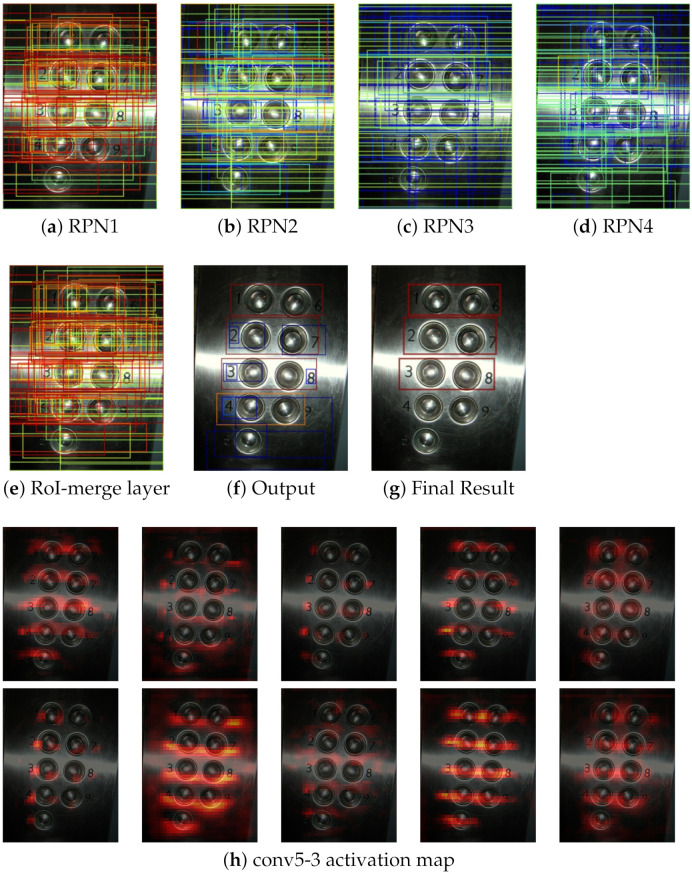
Failure example 2.

We analyzed the failure results of the proposed method. The failure examples are shown in Figure 8 and Figure 9a–e show the proposals from each RPN and RoI-merge layer, (f) is the outputs of the classification by the Fast R-CNN and non-maximum suppression, (g) shows the final output, and (h) is some examples of the output feature map from conv5-3.

Figure 8 shows some text regions in the bottom-right image were not detected. The RPNs generated proposals of all the text regions, as well as RoI-merge layer. However, proposals were misclassified. Thus, some proposals were rejected by low confidence as the final output. As shown in Figure 8h, the bottom-right text regions were not activated well. To correct the proposed method, the classifier in the proposed method needs more training. The total loss is mostly occupied by the RPN losses, in Equation (1). Thus, we need to take a balance over $L_{rpn_{i}}$ and $L_{fastrcnn}$.

Moreover, we discuss on the case of Figure 9. The results contained the digit regions, however, they included large background regions. There are some proposals fitted to only digits. However, such proposals were misclassified. On the other hand, proposals with large background regions were classified to text with high confidence. According to (h), background regions were activated. We can suppress the activation in the background by assigning more weight to the classifier.

## 5. Conclusions

We proposed a text detection method that is robust to text scales in natural scene images. The proposed method is based on the Faster R-CNN [3]. The main improvement is to introduce multiple RPNs to detect texts from different resolution feature maps. We designed the anchors suitable for Latin text detection by the analysis on the three datasets: COCO Text, Synth Text, and our dataset. We stress that these datasets are publicly and widely used in text detection studies. Thus, the proposed anchors ensure the generalization capability. The experimental results show that the proposed method outperformed the Faster R-CNN at F-score with more than 7 points. Moreover, the proposed method achieved comparable results to other methods. Therefore, we verified the effectiveness of the proposed method, especially for text scales.

## Figures and Tables

**Table 1:** Numerical results on ICDAR2013.

Method	Input Scale	Recall	Precision	F-Score	Time
Gupta+ [50]	Multiple	75.5	92.0	83.0	-
He+ [43]	Multiple	81	92	86	0.9 s
Liao+ [35]	Multiple	83	89	86	0.73 s
Tian+ [33]	Single	75.9	85.2	80.3	-
Zhong+ [37]	Single	83	87	85	1.7 s
Baseline Faster R-CNN [3]	Single	70.3	83	76.1	0.101 s
Proposed	Single	76.3	91.8	83.3	0.137 s
Proposed (competition mode)	Single	87.1	87.7	87.4	-

**Table 2:** Results on ablation study.

Method	Anchor	SSP-RPNs	Recall	Precision	F-Score	Time
Baseline			70.28	82.99	76.11	0.101
Anchor	✓		77.21	88.35	82.40	0.103
SSP-RPNs		✓	70.26	86.63	77.86	0.125
Proposed	✓	✓	76.29	91.81	83.33	0.137

## Data Availability

Publicly available datasets were analyzed in this study. This paper contains the links of the datasets.
